# A Novel 3D Visualized Operative Procedure in the Single-Stage Complete Repair With Unifocalization of Pulmonary Atresia With Ventricular Septal Defect and Major Aortopulmonary Collateral Arteries

**DOI:** 10.3389/fcvm.2022.836200

**Published:** 2022-04-25

**Authors:** Hailong Qiu, Shusheng Wen, Erchao Ji, Tianyu Chen, Xiaobing Liu, Xiaohua Li, Yun Teng, Yong Zhang, Rong Liufu, Jiawei Zhang, Xiaowei Xu, Jimei Chen, Meiping Huang, Jianzheng Cen, Jian Zhuang

**Affiliations:** ^1^Department of Cardiovascular Surgery, Guangdong Cardiovascular Institute, Guangdong Provincial People's Hospital, Guangdong Academy of Medical Sciences, Guangzhou, China; ^2^Laboratory of Artificial Intelligence and 3D Technologies for Cardiovascular Diseases, Guangdong Provincial Key Laboratory of South China Structural Heart Disease, Guangdong Provincial People's Hospital, Guangdong Academy of Medical Sciences, Guangzhou, China

**Keywords:** pulmonary atresia, ventricular septal defect, major aortopulmonary collateral arteries, three-dimensional printing, virtual reality, mixed reality, preoperative planning

## Abstract

**Objectives:**

Pulmonary atresia with ventricular septal defect and major aortopulmonary collateral arteries (PA/VSD/MAPCAs) is a relatively rare, complex, and heterogeneous congenital heart disease. As one of the effective treatments, the midline unifocalization strategy still remains complicated and challenging due to the diverse forms of MAPCAs and pulmonary arteries. The purpose of this study is to summarize our experience of a novel three-dimensional (3D) visualized operative procedure in the single-stage complete repair with unifocalization and to clarify the benefits it may bring to us.

**Methods:**

We described our experience of the 3D visualized operative procedure such as 3D printing, virtual reality (VR), and mixed reality (MR) technology in patients with PA/VSD/MAPCAs who underwent a single-stage complete repair with unifocalization. The data from the patients who underwent this procedure (3D group) and those who underwent the conventional procedure (conventional group) were compared.

**Results:**

The conventional and 3D groups included 11 patients from September 2011 to December 2017 and 9 from January 2018 to March 2021, respectively. The baseline characteristics such as age, body weight, preoperative saturation, the anatomy of the pulmonary arteries and MAPCAs, the Nakata index, and TNPAI had no statistical significance. All 9 patients in the 3D group were operated only through a median sternotomy, while 8 cases (72.7%) in the conventional group needed another posterolateral thoracotomy (*p* = 0.001). In the 3D group, the CPB time was shorter (93.2 ± 63.8 vs. 145.1 ± 68.4 min, *p* = 0.099), and the median pre-CPB time per MAPCAs was significantly shorter [25.7 (14.0, 46.3) vs. 65 (41.3, 75.0) min, *p* = 0.031]. There was no early death in the 3D group, while there were 3 in the conventional group (0 vs. 27.3%, *p* = 0.218).

**Conclusion:**

The novel 3D visualized operative procedure may help improve the performance of the single-stage complete repair with the midline unifocalization of PA/VSD/MAPCAs and help shorten the dissecting time of the MAPCAs. It may promote the routine and successful application of this strategy in more centers.

## Introduction

Pulmonary atresia with ventricular septal defect and major aortopulmonary collateral arteries (PA/VSD/MAPCAs) is a relatively rare, complex, and heterogeneous congenital heart disease. Although the treatment of this disease has made great progress in the past few decades, the optimal strategy still remains controversial with 2 antithetical extremes, the midline unifocalization strategy of the Stanford group (1) and the pulmonary rehabilitation strategy of the Melbourne group (2). The midline unifocalization approach was proposed by Reddy and colleagues in 1990s (3). The Stanford group has accrued the largest single-center experience in the world, indicating that the midline unifocalization strategy can lead to satisfactory surgical outcomes with 66% of the patients undergoing a single-stage complete repair with unifocalization (1). Sseveral other groups have also successfully performed the midline unifocalization operation with favorable results (4–7). However, due to the diverse forms of MAPCAs and pulmonary arteries, this operation still remains so complicated and challenging that it may be hard for other centers to accept it as a routine operation or perform it in the best way. In recent years, three-dimensional (3D) technologies such as 3D printing, virtual reality (VR), and mixed reality (MR) technologies have developed rapidly and have become more available. Since they all can provide an intuitive understanding of the anatomy, they have been used in the surgical treatment of some structural heart diseases and are considered effective (8–16). In our center, since January 2018, these 3D technologies were routinely used in the surgical treatment for patients with PA/VSD/MAPCAs as reported earlier (17), which we named the 3D visualized operative procedure. The purpose of this study is to summarize our experience about the 3D visualized operative procedure in the single-stage complete repair of PA/VSD/MAPCAs and to clarify the benefits it may bring to us by analyzing the data of these cases and the previous ones.

## Materials and Methods

The study was approved by the Guangdong Provincial People's Hospital Institutional Research Ethics Board (GDREC2019338H). All included patients were diagnosed with PA/VSD/MAPCAs by preoperative echocardiography and cardiac contrast-enhanced computed tomography (CT) scan and underwent the single-stage complete repair with unifocalization operation from September 2011 to March 2021. The included patients were divided into conventional and 3D groups based on different preoperative and intraoperative evaluation procedures, which included conventional operative and 3D visualized procedures at different time periods. Compare to the conventional operative procedure, the 3D visualized operative procedure added the 3D printing, VR, and MR technologies based on the 3D reconstruction of the CT data into the preoperative evaluation, surgical planning, and intraoperative navigation.

### Acquisition of 3D Printing, VR, and MR Models

The cardiac enhanced CT scan was performed in all patients before operation. Images were obtained using a 64-slice CT scanner or a 256-slice Multi-detector CT scanner in ECG-gated mode. The ECG-triggered data acquisition was set at 35–50% of the RR interval.

Under the guidance of senior radiologists, the modeling engineer used Mimics software (Materialize, Leuven, Belgium) to model the cardiovascular structures, the trachea, and the thorax based on the CT data, which were then imported into Materialize 3-matic research software (Materialize, Leuven, Belgium) to smooth the surface and hollow the inside. After setting each part to different colors, the 3D digital model was obtained in the STL file format. The 3D digital model was then imported into the VR and MR holographic systems developed by our laboratory, which can be used with the VR helmet (HTC, Taiwan, China) and the Hololens device (Microsoft, Washington, USA), respectively. In order to avoid the MAPCAs being obscured by other structures and save printing time and material, the 3D digital model for printing only contained the parts of the trachea, the aorta, the pulmonary arteries, and MAPCAs, with a designed base and some support columns between them, which was then imported into an integrated full-color 3D printer (J501 Pro, Sailner, Zhuhai, China) to print the 3D solid model.

### Conventional Operative Procedure

**Figure 1:**
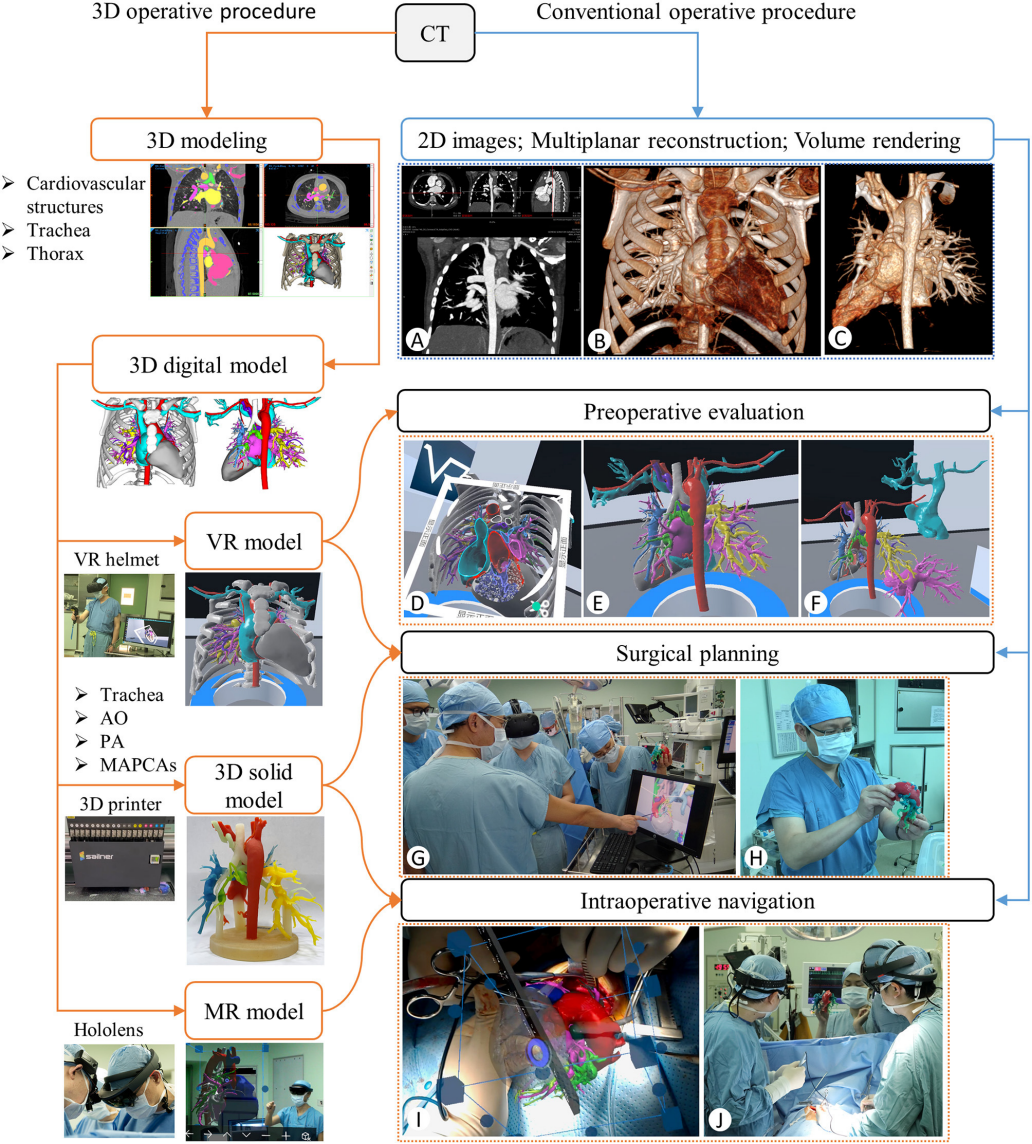
Flows of the 3D visualized (orange line) and the conventional (blue line) operative procedures. **(A)** CT Multiplanar Reconstruction to review the anatomy. **(B,C)** CT Volume rendering with and without the thorax part. **(D–F)** Preoperative evaluation with the section, rotation, and separation of the VR models. **(G,H)** Surgical planning with the VR and 3D solid models. **(I,J)** Determination of the MAPCAs location by coinciding the virtual and real hearts on the Hololens device **(I)** and anatomical review by observing the 3D solid model placed next to the operating table **(J)** during the operation.

Before the operation, the surgeons and radiologists read the cardiac CT images together to understand the anatomy of the pulmonary arteries and MAPCAs through multiplanar reconstruction (Figure 1A) and volume rendering (Figures 1B,C). At the same time, the surgeons memorized the key anatomical sites and planned the operation in mind. If any MAPCAs were originating from the descending aorta, we would first determine to dissect them from the left/right posterolateral thoracotomy.

During the operation, the MAPCAs originating from the descending aorta were fully dissected and identified with silk sutures through the posterolateral thoracotomy, which was then closed. Then, a median sternotomy was performed and the other MAPCAs were dissected. Once all MAPCAs were snared to achieve control, the cardiopulmonary bypass (CPB) was initiated followed by the rest of the operation. In order to measure the dissecting speed of the MAPCAs, we defined the pre-CPB time as the time between the beginning of the operation and CPB and the pre-CPB time per MAPCAs as the ratio of the pre-CPB time to the number of MAPCAs.

### 3D Visualized Operative Procedure

Before the operation, the VR model was used to understand the anatomy of all the cardiovascular structures in the whole thoracic cavity, especially the blood supply range of pulmonary arteries and MAPCAs, and their adjacent relationship with other structures (Figures 1D–F). All the anatomical structures on the VR model were used to plan the best dissecting path and the reconstruction plan (Figure 1G). At the same time, the actual size of the pulmonary arteries, MAPCAs, the aorta, and the trachea and the distance between them were observed by the 3D solid model (Figure 1H).

During the operation, after the median sternotomy was performed, we coincided the virtual heart with the real heart as far as possible to further determine the location of the MAPCAs of the descending aorta with the Hololens (Figure 1I). Furthermore, the 3D solid model was placed next to the operating table so that the surgeon could observe it to review the anatomy of the pulmonary arteries and MAPCAs when confused (Figure 1J). Once all MAPCAs were snared to achieve control, the CPB would be initiated, followed by the rest of the operation.

### Statistical Analysis

All data were analyzed with IBM SPSS v26.0 (IBM, Armonk, NY). Normally and non-normally distributed continuous variables are reported as mean ± standard deviation (*SD*) and median [interquartile range (IQR)]. To compare differences between groups, the Student *t*-test and the Mann–Whitney U-test were used for normally and non-normally distributed data whereas the chi-squared test was used for categorical variables. All tests were two-tailed, and a *p* < 0.05 was considered statistically significant.

## Results

### Baseline Characteristics

The conventional and 3D groups included 11 patients from September 2011 to December 2017 and 9 patients from January 2018 to March 2021, respectively. The mean age and body weight at operation in the 3D group were more than those in the conventional group but without statistical significance (55.6 ± 39.0 vs. 38.4 ± 51.0 months, *p* = 0.419; 13.3 ± 5.8 vs. 10.8 ± 6.9 kg, *p* = 0.252). There was no significant difference in the median preoperative hemoglobin saturation (SaO_2_) between the 3D and conventional groups [88 (76.89) vs. 84 (80.89)]%, *p* = 0.955]. The situation of absent main/left native/right native pulmonary arteries and non-confluent pulmonary artery were similar between the two groups. There were 29 and 28 MAPCAs in 3D and conventional groups, respectively, with the mean number per patient of 3.2 ± 1.4 and 2.5 ± 1.0, respectively, which were not significantly different. The median Nakata index and the mean TNPAI of 3D groups were both higher than those of the conventional group but without statistical significance [121.4 (60.2, 148.2) vs. 51.9 (0, 145.5) mm^2^/m^2^, *p* = 0.452; 322.1 ± 156.9 vs. 245.8 ± 165.4 mm^2^/m^2^, *p* = 0.308]. The baseline characteristics are summarized in Table 1.

**Table 1:** Preoperative characteristics.

**Variable**	**3D Group (*n* = 9)**	**CT Group (*n* = 11)**	***p*-value**
Age at operation (months)	55.6 ± 39.0	38.4 ± 51.0	0.419
Gender (male/female)	4/5	4/7	1.000
Weight (kg)	13.3 ± 5.8	10.8 ± 6.9	0.252
SaO_2_ before operation (%)	88 [76, 89]	84 [80, 89]	0.955
Pulmonary arterial anatomy (*N*, %)			
Absent main pulmonary artery	7 (77.8%)	7 (63.6%)	0.642
Non-confluent pulmonary arteries	4 (44.4%)	6 (54.5%)	1.000
Absent left native pulmonary artery	4 (44.4%)	6 (54.5%)	1.000
Absent right native pulmonary artery	1 (11.1%)	4 (36.4%)	0.319
MAPCAs anatomy			
Total number	29	28	
Number per patient	3.2 ± 1.4	2.5 ± 1.0	0.229
Nakata index of native PA (mm^2^/m^2^)	121.4 [60.2, 148.2]	51.9 [0, 145.5]	0.452
Total neopulmonary artery index (mm^2^/m^2^)	322.1 ± 156.9	245.8 ± 165.4	0.308

### Surgical Procedures

All 9 patients in the 3D group were operated only through a median sternotomy, while 8 cases (72.7%) needed another posterolateral thoracotomy in the conventional group, which was statistically different (*p* = 0.001). The median number of recruited MAPCAs in the 3D and the conventional groups were 2.6 and 1.7, respectively, without statistical significance (*p* = 0.106). There was no difference in CPB, ACC, and operation time. The mean pre-CPB time was smaller in the 3D group but without statistical significance (93.2 ± 63.8 vs. 145.1 ± 68.4 min, *p* = 0.099). However, when the number of MAPCAs was taken into consideration and divided into the pre-CPB time, the median pre-CPB time per MAPCAs was significantly smaller in the 3D group [25.7 (14.0, 46.3) vs. 65 (41.3, 75.0) min, *p* = 0.031]. The operative details are summarized in Table 2.

**Table 2:** Operative details and early outcomes.

**Variable**	**3D Group (*n* = 9)**	**CT Group (*n* = 11)**	***p*-value**
Operative details			
Posterolateral thoracotomy (*N*, %)	0 (0%)	8 (72.7%)	0.001
Total number of recruited MAPCAs	23	19	
Number of recruited MAPCAs per patient	2.6	1.7	0.106
Cardiopulmonary bypass time (min)	247 ± 76	212 ± 71	0.304
Aortic cross-clamp time (min)	99 ± 43	126 ± 43	0.183
Operation time (hour)	7.1 ± 2.7	7.7 ± 2.8	0.647
Pre-CPB time (min)	93.2 ± 63.8	145.1 ± 68.4	0.099
Pre-CPB time per MAPCAs (min)	25.7 [14.0, 46.3]	65 [41.3, 75.0]	0.031
Mechanical ventilation time (day)	5.2 [1.3, 9.8]	5.5 [1.0, 13.6]	0.815
ICU stay (day)	6.7 [3.8, 11.1]	8.2 [5.7, 14.1]	0.481
Hospital stay after operation (day)	23.0 [13.0, 26.5]	17.0 [14.2, 31.0]	0.869
SaO_2_ at dicharged (%)	98 [98, 98]	95 [94.25, 98]	0.203
Early mortality (*N*, %)	0 (0%)	3 (27.3%)	0.218

### Early Outcomes

The median postoperative mechanical ventilation time, SaO_2_ at discharge, and postoperative hospital stay were similar between the two groups [5.2 (1.3, 9.8) vs. 5.5 (1.0, 13.6) days, *p* = 0.815; 98 (98.98) vs. 95 (94.25, 98) %, *p* = 0.203; 23.0 (13.0, 26.5) vs. 17.0 (14.2, 31.0) days, *p* = 0.869]. In the 3D group, the median ICU stay time was shorter but without statistical significance [6.7 (3.8, 11.1) vs. 8.2 (5.7, 14.1) days, *p* = 0.481]. There was no early death in the 3D group, while there were 3 in the conventional group (0 vs. 27.3 %, *p* = 0.218). Early outcomes are summarized in Table 2.

## Discussion

This study reviewed our application experience of the 3D visualized operative procedure in 9 patients with PA/VSD/MAPCAs who underwent single-stage complete repair with midline unifocalization. Compared to the previous 11 cases, the early results of these 9 cases were satisfying, as the data showed that the 3D visualized operative procedure helped us to avoid the lateral thoracotomy and shorten the dissecting time of MAPCAs.

### 3D Printing, VR, and MR Models

Since January 2018, our center has been using the 3D visualized operative procedure for the surgical treatment of many complex structural heart diseases (SHDs). Although the 2020 ESC Guidelines for the management of adult congenital heart disease mentioned that “3D CCT and CMR reconstructions can also be used for virtual reality rehearsal of interventions or planning from patient-specific 3D prints” (18), our center may be the first one in the world to systematically apply these technologies in clinical practice as there are few similar reports that we could find till date. PA/VSD/MAPCAs became one of the beneficial SHDs due to its complex anatomy.

Different from only using the 3D printed model reported by Ryan et al. (19), our 3D visualized operative procedure is to introduce 3D printing, VR, and MR models into different treatment processes of PA/VSD/MAPCAs according to their technical characteristics. To be more specific, the VR model can intuitively display all the anatomical structures with full depth perception (8). The VR platform developed by our center allowed rotation, movement, scaling, section, and notes drawing of the VR model, which took little time and effort to be converted from the 3D digital model. We used it to comprehend all the anatomical information of the cardiovascular structures, the trachea, and the thorax and to plan the details of MAPCA dissection and the unifocalization operation.

Compared with the VR model, the size of the 3D solid model was closer to the real structures but it cost more time and material for printing, which was relatively expensive. Therefore, we only printed few major structures such as the pulmonary arteries, MAPCAs, the trachea, and the aorta to reconfirm their size and the relationship between them as well as the feasibility of the planned surgical details. Considering the surgeon's inconveniences of wearing the VR/MR devices during the operation, the 3D solid model was also placed next to the operating table for the surgeons' to review the anatomy.

As for the MR model in the Hololens, its most prominent characteristic was that the virtual structures could coincide with the real structures to reveal the covered parts, like the “X-ray vision” but without extra radiation. After the median thoracotomy was performed, we used the ascending aorta and the myocardium as anchor points to coincide the virtual and real cardiovascular structures to specify the location and course of the deep MAPCAs. However, since the virtual structures would interfere with the vision of the real ones and the Hololens was relatively heavy, it could not be worn throughout the operation, which needs to be further improved in hardware and software.

### Value of the 3D Visualized Operative Procedure

The 3D visualized operative procedure may contribute to reducing the difficulty of the midline unifocalization operation and promoting its routine and successful performance in more centers. The unifocalization operation for PA/VSD/MAPCAs is still complex and challenging. First, MAPCAs can originate from various positions, such as the ascending/descending aorta, the aortic arch, the subclavian artery, and the coronary artery, and some of them are tortuous (6, 7). Second, the wall of MAPCAs is relatively thin and fragile, so it is easy to be broken but hard to fix. Third, it is imperative to perform the entire surgical dissection before the administration of heparin to achieve complete hemostasis and the beginning of the CPB in order to avoid the intraparenchymal hemorrhage and excessive pump flow from the MAPCAs to the lung (20).

Although this operation is routine and has achieved good results in some groups, such as the Stanford group (1), the Birmingham group (4, 5), the Toronto group (7, 21), and the Rome group (6), the above complexities still lead very few other groups to routinely and skillfully perform it, especially in developing countries like China. However, in developing countries, the age of the first treatment for patients with PA/VSD/MAPCAs is usually older (22, 23); thus, the best time to conduct the rehabilitation strategy may have passed and they are more in need of the midline unifocalization strategy. In the past, since many patients had some MAPCAs originating from the descending aorta, the dissection of these MAPCAs was completed through the posterolateral thoracotomy approach for the sake of safety, and the median sternotomy was then performed to complete the dissection of other MAPCAs and the rest of the operation in our center and some other centers (24). In addition to causing two incisions, since it was difficult for surgeons to fully remember the anatomical information of all structures, this operation also wasted more time on hesitation during dissection of the MAPCAs and the review of the CT images. However, by following the 3D visualized operative procedure, surgeons could always keep familiar with the anatomical information of all the important structures and the detailed preoperative plan. This study showed that the pre-CPB time and the pre-CPB time per MAPCAs were shorter with the application of the 3D visualized operative procedure, which means that we can quickly dissect all the MAPCAs through the median sternotomy in the safe way planned preoperatively without the posterolateral thoracotomy. In addition, there was no death in the 3D group, which may benefit from adequate preoperative assessment and the better reconstruction of the new pulmonary arteries. These improvements implied that the 3D visualized operative procedure may contribute to reducing the difficulty of the midline unifocalization operation in our center, which may also be effective in other centers.

### Advantages and Challenges of 3D Technologies

There are obvious advantages in the application of 3D technologies in complex SHDs like PA/VSD/MAPCAs. First, since these technologies are based on CT, magnetic resonance imaging (MRI), and 3D echocardiography, they lead to neither more examinations nor additional damages. In addition, compared with the traditional imaging modalities, 3D technologies can display the structures more intuitively and improve the efficiency and quality of the surgeon's understanding of the anatomy and even change the surgical approach (12). It seems beneficial for all surgeons and may be more preferred among the younger ones (25), who may not have so much surgical experience.

However, there are some challenges waiting to be solved in the clinical application of 3D technologies. First of all, the extra cost of the professional software and hardware need to be solved, such as modeling software, the 3D printer and materials, and the VR and MR software. They may be paid by the technology companies or hospitals at first but by the patients themselves eventually. Second, the training of the 3D technical engineer needs to be solved. It mainly includes 3D modeling engineers, 3D printing model post-processing engineers, and software and hardware maintenance, which are relatively scarce at present. Third, the process of 3D printing still takes a long time, from several hours to several days depending on the model size. It has to be faster to meet the clinical needs. In addition, the physical parameters of the printed model are far from the real structure, which limits its application in surgical simulation and training. Furthermore, there is currently no standard 3D visualized operative procedure that suits all kinds of SHDs. Therefore, each SHD needs an individual 3D visualized operative procedure, which should be developed by the surgeons and engineers together. In face of the above challenges, our experience is to set up a special cooperation platform, which can be a joint laboratory or department. With this cooperation platform, on the one hand, the clinical needs can be fed back to the companies or institutions for the adaptation and development of new 3D technologies (26–28); on the other hand, the existing new technologies can be extended to clinical applications, so that they can be accessible for more surgeons.

Different from our center using cardiac CT, cardiac catheterization is still currently used to determine the anatomy of the cardiovascular structures and bronchopulmonary segment perfusion for patients with PA/VSD/MAPCAs in many centers. However, according to our previous study, through volume rendering and multiplanar reconstruction, low-dose cardiac CT can also clearly display the cardiac anatomy in congenital heart disease (29). Therefore, we only performed cardiac CT for the majority of the untreated patients with PA/VSD/MAPCAs in recent years. The unique advantage of cardiac CT is that it can provide data with a high spatial resolution for 3D reconstruction, which is the basis for the application of all novel 3D technologies. Note that cardiac catheterization is still a very useful modality for postoperative patients because the surgeon can directly evaluate the anatomy and pressure difference of the stenoses in the right ventricular and pulmonary arteries and branches and perform balloon dilation simultaneously, if necessary.

## Limitations

There are several limitations to our research. (1) This study is a single-center retrospective study, and the number of cases included is relatively small. In the future, our center will continue to accumulate more cases and actively promote the 3D visualized operative procedure to other centers. (2) The operation periods of the two groups are different, which means that there may be a technical level bias and other relevant biases. (3) PA/VSD/MAPCAs is complex and the difference in anatomical heterogeneity between the two groups is difficult to completely quantify, which may lead to statistical bias, although it is a common problem faced by all researchers. In future, as the amount of data becomes larger, we will be able to include more quantitative indicators and adopt the method of propensity score matching to reduce this bias.

## Conclusion

The novel 3D visualized operative procedure may help improve the performance of the single-stage complete repair with the midline unifocalization of PA/VSD/MAPCAs and shorten the dissecting time of the MAPCAs. It may promote the routine and successful application of this strategy in more centers.

## Data Availability Statement

The raw data supporting the conclusions of this article will be made available by the authors, without undue reservation.

## Ethics Statement

The studies involving human participants were reviewed and approved by Guangdong Provincial People's Hospital Institutional Research Ethics Board. Written informed consent to participate in this study was provided by the participants' legal guardian/next of kin. Written informed consent was obtained from the individual(s) for the publication of any potentially identifiable images or data included in this article.

## Author Contributions

HQ, SW, and EJ conducted data collection and statistical analysis and wrote the manuscript. MH and JCe conducted data validation and revised the manuscript. JZhu provided funding support and supervision. TC, XLiu, XLi, YT, YZ, RL, JZha, XX, and JCh revised the manuscript. All authors contributed to the article and approved the submitted version.

## Funding

This work was supported by the Science and Technology Planning Project of Guangdong Province (2019B020230003, 2018B090944002, and 2020B1111170011), Guangdong peak project (DFJH201802), National Key Research and Development Program (2018YFC100168), and the National Natural Science Foundation of China (62006050).

## Conflict of Interest

The authors declare that the research was conducted in the absence of any commercial or financial relationships that could be construed as a potential conflict of interest.

## Publisher's Note

All claims expressed in this article are solely those of the authors and do not necessarily represent those of their affiliated organizations, or those of the publisher, the editors and the reviewers. Any product that may be evaluated in this article, or claim that may be made by its manufacturer, is not guaranteed or endorsed by the publisher.
